# Psychosis and Personality Changes Following Traumatic Brain Injury

**DOI:** 10.7759/cureus.72849

**Published:** 2024-11-01

**Authors:** David Hanna, Sonya Priven, Nathan Carroll, Heba Ekladios, Adriana Fitzsimmons

**Affiliations:** 1 Psychiatry, Hackensack Meridian School of Medicine, Nutley, USA; 2 Psychiatry, Rowan-Virtua School of Osteopathic Medicine, Stratford, USA; 3 Psychiatry, Hackensack Meridian Jersey Shore University Medical Center, Neptune, USA

**Keywords:** frontal convexity, personality changes, psychosis, schizophrenia, tbi

## Abstract

Psychotic disorders following traumatic brain injury (TBI) are a significant concern in psychiatry, with chronic sequelae often overlooked. The pathophysiology of post-TBI psychosis involves neuroplasticity and neuronal remodeling, potentially exacerbating vulnerabilities to chronic psychotic disorders. Differentiating post-TBI psychosis from primary schizophrenia and other psychotic conditions is crucial for appropriate treatment. This case report discusses a 63-year-old male with a history of TBI from a gunshot wound sustained in adolescence, who later developed schizophrenia. The report explores the patient's psychiatric symptoms, cognitive dysfunction, personality changes, and imaging findings. The report examined the complex relationship between TBI and psychotic symptoms, considering genetic, neurobiological, and psychosocial factors. The potential for TBI to increase the risk of psychotic conditions, especially in genetically predisposed individuals, was discussed. The challenges in establishing a direct causal link between TBI and schizophrenia were addressed.

## Introduction

Psychotic symptoms and disorders following traumatic brain injury (TBI) are not uncommon [1]. Acute effects of TBI include confusion, agitation, and delirium. Chronic sequelae are often overlooked and may take the form of changes in cognition, memory, perception, language, intelligence, personality change, mood disorders, or psychoses [2]. The pathophysiology of these latter effects is thought to involve neuroplasticity. It is hypothesized that TBIs lead to neuronal remodeling, which could cause impairments in individuals already at risk of developing schizophrenia [3]. The development of positive psychotic symptoms following TBI has been shown to be correlated with hippocampal changes, particularly volume loss, that lead to dopamine circuit dysregulation - a process known to be implicated in schizophrenia [4]. Schizophrenia is a severe psychiatric disorder classified as two or more of the following symptoms: delusions, hallucinations, disorganized speech, grossly disorganized behavior, and negative symptoms, including diminished emotional expression or avolition.

Today, schizophrenia affects approximately 1% of people worldwide and is a persistent, often devastating mental illness [5]. The etiology of schizophrenia is still indefinite despite a century of research, and its pathogenesis appears to involve the interplay of biological theories and family psychosocial theories. A potentially large number of genes play a role in the biological hypothesis of schizophrenia. The neurobiology of schizophrenia is complex. Genetic factor is a major contributing factor in developing symptoms of schizophrenia. However, environmental factors could be a potential modulating factor in the expression of symptoms [6]. Neurotransmitters and neuroanatomy also play a role in developing schizophrenia. Dopamine plays a major role in psychosis. In addition, there are different neurotransmitter disturbances, neuronal disconnectivity, neurotoxicity, and neuroinflammation involved in its pathogenesis. Pathologic family dynamics and environmental stressors contribute to the development of schizophrenia [7].

A higher prevalence of positive symptoms and focal and temporal abnormalities found on neurological exams are key in differentiating between post-TBI psychosis and schizophrenia [8]. A case-controlled study showed that positive psychosis is predominant in head injury-related psychosis (schizophrenia) with persecutory delusion and auditory hallucination. Only 22.2% of patients demonstrated negative symptoms, including flat affect, abolition, and asociality [9]. Predictors of personality change following TBI include lesions in the frontal white lobe matter, superior frontal gyrus, or orbital gyrus [10], as well as severity of TBI, prior history of psychiatric illness [11], and lower socioeconomic status [12]. Lesions to the aforementioned areas of the brain have also been strongly associated with aggressive behavior and impulsiveness following TBI [13]. The majority of reported cases of post-TBI psychotic disorder have been males, with symptom onset within two years after moderate to severe head injury, imaging showing localization to the frontal and temporal areas, and predominantly positive symptomatology consisting of delusions and hallucinations [14]. The Brain Injury Personality Scale has been developed in order to classify and measure the severity of personality change following TBI [15].

In this review, we discuss a strategy for determining how particular common and core clinical features of the illness are associated with pathophysiology in certain circuits of the cerebral cortex.

## Case presentation

This is a 63-year-old male with a past medical history of hypertension, chronic kidney disease on dialysis three times a week, and TBI who presented to the outpatient office for continuation of care. Of note, he was shot in the head with a bullet through his right frontal bone area at 18 years old. Per sister, the patient, before his injury, used to be very social and smart and had a lot of friends. Over several years following the trauma, he was diagnosed with schizophrenia. He experienced progressive auditory hallucinations and paranoid delusions. In addition, he suffered from personality changes, including impulsivity, “excessive spending of money in gambling and in buying unnecessary things,” and the development of a short temper. TBI was also complicated by significant cognitive dysfunction and seizures. The patient has a history of inpatient admission in a psychiatry unit in the setting of decompensated mental illness. He has no family history of schizophrenia or psychosis. The patient has been adherent to risperidone 3 mg for psychosis and sertraline 50 mg for mood. At this time, he lives alone and is able to perform all his activities of daily living such as feeding, dressing, and bathing.

In the clinic, he presented with a history of depressed mood, aggressive behaviors, and suicidal ideation and was internally preoccupied with disorganized thoughts at the time of the visit.

Differential diagnoses include psychosis secondary to TBI, frontotemporal dementia, chronic traumatic encephalopathy, and substance-induced psychosis, all of which can account for the impulsivity and aggression seen in this patient.

**Figure 1:**
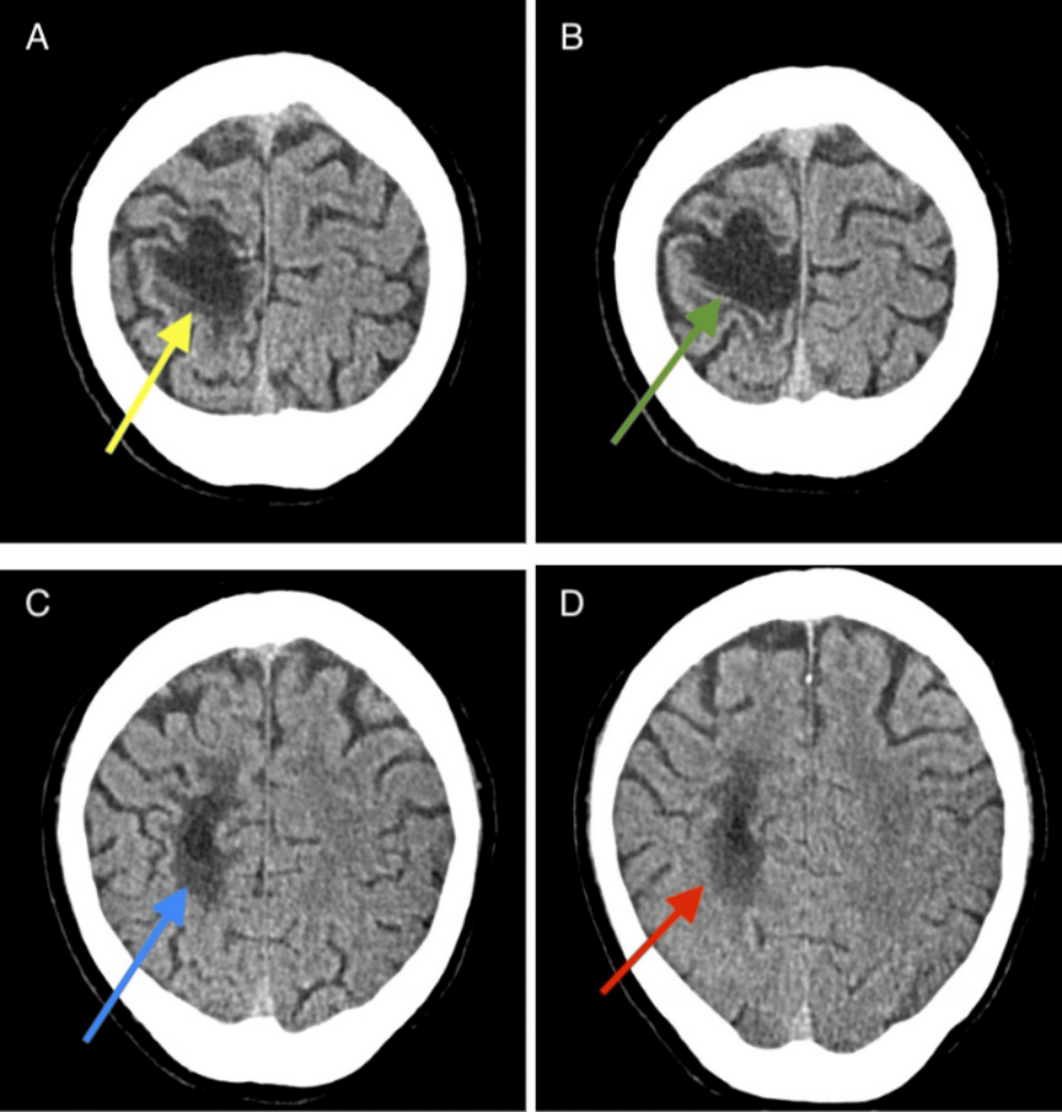
CT head without contrast Non-contrast axial CT scan of the head demonstrating a deformity in the calvarium just above the right frontal convexity region denoted by yellow and green arrows (A, B), suggesting previous trauma. Additionally, encephalomalacia in the right frontal convexity region is denoted by blue and red arrows (C, D), characterized by an area of low attenuation consistent with loss of brain tissue and gliosis. No acute hemorrhage or mass effect is present.

CT head without contrast revealed an area of encephalomalacia in the right frontal convexity region (Figures 1C-1D), as well as a deformity in the calvarium just above this region (Figures 1A-1B).

## Discussion

The above case describes a 63-year-old patient who developed behavioral changes and progressive psychosis in the context of TBI and right frontal lobe injury. This case adds to the literature on the association between TBI and resulting psychosis and/or personality changes.

Psychosis is an uncommon but devastating sequelae of TBI. The onset of TBI and the reported occurrence of psychosis is variable, and it ranges from two days and 48 years [16].

It is known that TBI raises the risk for a variety of neuropsychiatric disorders. However, the question of whether TBI is a risk factor for schizophrenia or psychosis remains somewhat controversial. TBI appears to have a larger impact on people who are genetically predisposed to schizophrenia [17]. In addition, Fujii supported the general hypothesis that a pre-existing neurological condition is a risk factor for developing psychosis [8].

Research derived from case studies, medical reports from World War II, and modern structural imaging data indicates that frontal and temporal brain damage is linked to psychosis resulting from TBI [8].

Malaspina revealed that TBI doubled the risk of schizophrenia in family members of relatives of individuals with schizophrenia. However, she also noted that TBI was more prevalent in relatives than in relatives of control denoting that schizophrenia genes may impact both the exposure to and consequences of TBI [17]. Furthermore, Fann et al. [11] found a greater prevalence of preexisting psychosis in people who suffered head injuries, leading them to hypothesize that psychosis increases the risk of TBI. Examining the relationship between TBI and schizophrenia is made more challenging by the apparent complexity of the causal pathway.

## Conclusions

Psychosis is a severe mental health condition characterized by a disconnection from reality. Individuals experiencing psychosis may have hallucinations, delusions, and disorganized thinking. Psychosis can also result in disorganized behavior and an inability to distinguish what is real from what is not, significantly affecting a person's ability to function in daily life. It can occur as part of other psychiatric disorders, such as schizophrenia, a bipolar disorder, or as a result of substance abuse, brain injuries, or severe stress. TBI refers to brain damage caused by an external physical force, such as a blow to the head from accidents, falls, or violence. The severity of TBI can range from mild concussions to severe brain injuries, which can result in long-term impairments in cognitive function, emotional regulation, and behavioral control. TBI has also been strongly linked to an increased risk of developing psychosis. Studies indicate that individuals with a history of moderate to severe TBI have a 60% greater risk of experiencing psychotic symptoms compared to those without such injuries. These symptoms may include hallucinations, delusions, and significant behavioral changes.

This case report contributes to the existing body of literature that explores the relationship between TBI and subsequent psychosis. It suggests that brain trauma may disrupt critical neural circuits - particularly those involved in dopamine regulation and frontal-limbic brain networks - that are essential for maintaining cognitive control and emotional regulation. As a result, individuals who suffer from a TBI may become more vulnerable to developing psychotic disorders. However, while there is clear evidence of a strong association between TBI and the onset of psychosis, causality has not been definitively established. The relationship is likely influenced by multiple factors, including genetic predisposition, pre-existing mental health conditions, and environmental triggers. Further research is necessary to determine whether TBI directly contributes to the onset of psychosis or whether it acts as a trigger in individuals already predisposed to psychiatric disorders.
